# The Role of T Follicular Helper Cells and T Follicular Regulatory Cells in the Pathogenesis of Autoimmune Hemolytic Anemia

**DOI:** 10.1038/s41598-019-56365-3

**Published:** 2019-12-24

**Authors:** Yuhan Gao, Haiqiang Jin, Ding Nan, Weiwei Yu, Jianhua Zhang, Ying Yang, Ruiqin Hou, Ranran Qin, Hongjun Hao, Yongan Sun, Wenqin Tian

**Affiliations:** 1 Department of Blood Transfusion, Peking University Second Hospital, Beijing, China; 2 Department of Neurology, Peking University First Hospital, Beijing, China

**Keywords:** Cytokines, Immunopathogenesis

## Abstract

Autoimmune hemolytic anemia (AIHA) is an acquired autoimmune disease mediated by antibodies against the patient’s red blood cells. However, the underlying mechanisms for antibody production are not fully understood. Previous studies of etiology and pathogenesis of AIHA mainly focus on autoreactive B cells that have escaped tolerance mechanisms. Few studies have reported the function of T_FH_ and T_FR_ cells in the process of AIHA. The present study aimed to explore the potential mechanism of T_FH_ and T_FR_ cells in the pathogenesis of AIHA. With the model of murine AIHA, increased ratios of T_FH_:T_FR_, elevated serum IL-21 and IL-6 levels, and upregulated Bcl-6 and c-Maf expression were reported. Also, adoptive transfer of purified CD4^+^CXCR5^+^CD25^-^ T cells from immunized mice promoted the induction of autoantibody in the AIHA mouse model. Altogether, our data demonstrate the important role of T_FH_ cells for control and induction of AIHA. In the light of the key contributions of T_FH_ cells to the immune response in AIHA, strategies aimed at inhibiting the T_FH_ development or function should be emphasized.

## Introduction

Autoimmune hemolytic anemia (AIHA) is an acquired autoimmune disease resulting in the production of antibodies directed against the patient’s red blood cells (RBCs) causing shortened erythrocyte lifespan^1–3^. The most common form of AIHA is warm AIHA characterized by the presence of warm-type autoantibodies—immunoglobulin G (IgG) which reacts optimally at 37 °C, causing RBC extravascular destruction by tissue macrophages^4,5^. The main treatment of AIHA includes RBC transfusion and immune system inhibitors such as corticosteroids. Transfusion of RBC in AIHA patients is challenging as the autoantibodies in the patients are often reactive to the transfused RBCs, making every unit of blood incompatible. Moreover, the relapse rate is as high as 50% in patients refractory to steroids^6–8^. Thus, there is an urgent need to understand the mechanism of autoantibody production in AIHA so that better therapies can be designed.

Previous studies of the etiology and pathogenesis of AIHA have focused on the autoreactive B cells that have escaped tolerance mechanisms and regulatory T cells (Treg)^9^. Few studies have reported the function of T follicular helper cells (T_FH_) and T follicular regulatory cells (T_FR_) in the process of AIHA. A highly specialized CD4^+^ T cell subpopulation, T_FH_, has recently received immense attention, as they play important role in the regulation of germinal center (GC) reactions and antibody production. T_FH_ cells are characterized by the expression of the transcription factor the nuclear transcriptional repressor B cell lymphoma 6 (Bcl-6), the chemokine receptor chemokine (C-X-C motif) receptor 5 (CXCR5), inducible co-stimulator (ICOS), programmed cell death protein-1(PD-1), and production of high levels of interleukin 21 (IL-21)^10–13^. Of the cytokine signaling, interleukin 6 (IL-6) and IL-21 play a critical role in T_FH_ differentiation and function maintenance because of the upregulation of Bcl-6 and CXCR5 expression through signal transducer and activator of transcription 3 (STAT3)^14–17^. The main functions of T_FH_ cells are to support GC formation and reactions, provide B cells with essential maturation signals, drive antibody class switching, govern the generation of high-affinity antibodies, and promote memory formation^13,18–20^.

T_FR_ cells represent a highly specialized subpopulation of Foxp3^+^ Tregs that co-express T_FH_ features, such as Bcl-6, CXCR5, ICOS, PD-1 and Treg features CD25 and Foxp3^21^. T_FR_ cells have the ability to inhibit T_FH_ activation and cytokines production and suppress B cell GL7 and B7-1 expression and limited class switch recombination occurring in the GC via high expression of cytotoxic T-lymphocyte-associated protein 4 (CTLA-4) and production of inhibitory cytokine— interleukin 10 (IL-10) and transforming growth factorβ (TGFβ)^22,23^. The involvement of T_FR_ cells in the pathogenesis of human autoimmune diseases remains speculative, but an alteration of the T_FR_:T_FH_ ratio is observed in the blood of patients suffering from several autoimmune diseases, such as child immune thrombocytopenia^24^, and rheumatoid arthritis^25^.

Considering over-activation of B cells and overproduction of autoantibodies, we hypothesize T_FH_ and T_FR_ cells play a vital role in the process of AIHA. Here, we utilize the murine AIHA model to determine the role of T_FH_ and T_FR_ for the induction of AIHA. Our research has demonstrated that there is an increased ratio of T_FH_:T_FR_, elevated serum IL-21 and IL-6 levels, and upregulated Bcl-6 and c-Maf expression at the transcriptive levels in autoantibody-positive AIHA mouse. In addition, adoptive transfer of purified CD4^+^CXCR5^+^CD25^−^ T cells, but not CD4^+^CXCR5^-^CD25^−^ T cells, from immunized mice promoted the induction of autoantibody in the AIHA mouse model. Altogether, our data demonstrate the important role of T_FH_ cells for the control and induction of AIHA. In the light of the key contributions of T_FH_ cells to the immune response in AIHA, strategies aimed at inhibiting the T_FH_ development or function should be emphasized for the treatment of AIHA.

## Results

### Expression of CD4^+^CXCR5^+^CD25^−^ T_FH_ cells in AIHA mouse model

**Figure 1:**
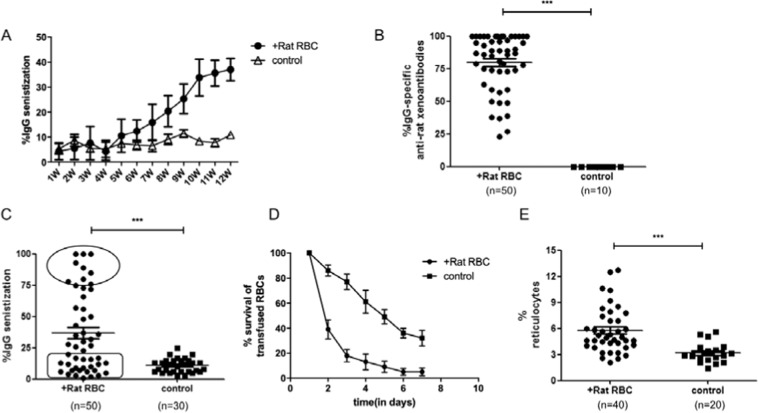
Development of the AIHA mouse model. Female C57BL/6J mice aged 8-10 weeks were immunized on a weekly basis with rat RBCsfor 12 weeks. (**A**) Level of IgG-specific autoantibodies on mouse RBCs was measured by flow cytometry on the day before rat RBCs injection and expressed as percentage of background unstained cells. This experiment was repeated three times with at least 10 mice for each group each time. (**B**) Levels of IgG-specific anti–rat xenoantibodies in plasma were measured by first incubating rat erythrocytes with diluted mouse plasma followed by staining with FITC-conjugated anti–mouse IgG. The analysis was performed by flow cytometry and the percentage of rat RBCs that have antibodies bound to them is shown on the Y-axis. (**C**) Theexpression of IgG-specific autoantibodies on mouse RBCs at week 12. Each plot represents one mouse in each group. The oval box represents the cohort with the highest levels of autoantibodies, whereas the square box includes the group with background control levels of autoantibodies. (**D**) Red cell survival studies were performed using PKH-26 labeled C57BL/6J mouse RBCs transfused into mice immunized with rat RBCs or PBS as a negative control.At times indicated, venous blood was sampled and analyzed by flow cytometry for the fraction of fluorescent RBCs. To show the RBCs clearance kinetics, detectable RBCs at 1 minute after injection were defined as 100%, and the remaining RBCs were calculated at different time points by divided the total RBCs. This experiment was repeated three times with two mice in each group. (**E**) Numbers of circulating reticulocytes in mice expressed as a percentage. Data shown were the mean ± SEM. The horizontal lines show the median.

To study the role of T_FH_ in AIHA, an AIHA mouse model was constructed according to the method described previously^26,27^. In our model, erythrocyte autoantibodies were detectable within 5-6 weeks and constantly increased in the following six weeks. In the twelfth week, nearly all the mice developed rat RBC-specific xenoantibodies, and approximately 40% of mice developed AIHA, as evidenced by the presence of red cell-specific autoantibodies on their RBCs, increased destruction of transfused mouse RBCs, and increased levels of circulating reticulocytes (Fig. 1A–E).

**Figure 2:**
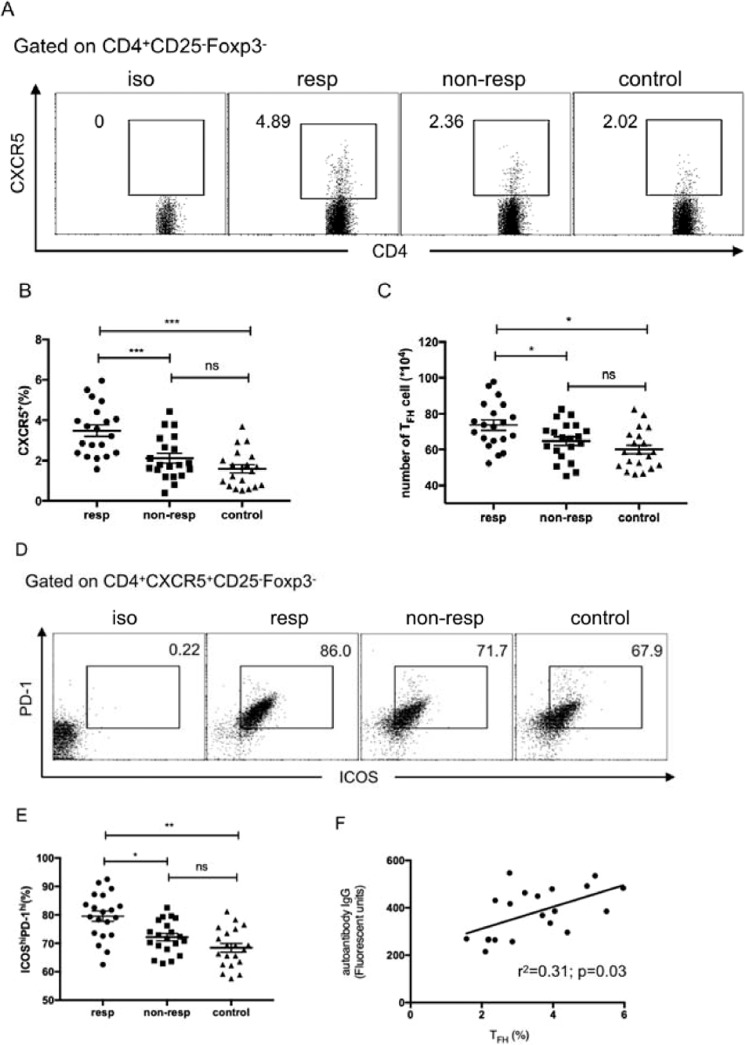
Increased T_FH_ cells in autoantibody-positive AIHA mice. (**A**) The expression of CXCR5 in spleen CD4^+^CD25^−^FoxP3^−^T cells from the responder, non-responder, and control groups. (**B**) The percentages of CXCR5^+^ cells in CD4^+^CD25^−^FoxP3^−^ cells in these three groups. (**C**) The number of CD4^+^CXCR5^+^ CD25^−^FoxP3^−^T_FH_ cells in spleen in these three groups. (**D**) The expression of ICOS and PD-1 in CD4^+^CXCR5^+^ CD25^−^FoxP3^−^T_FH_ cells from these three groups. (**E**) The percentages of ICOS^hi^PD-1^hi^ cells in spleen CXCR5^+^CD4^+^CD25^−^FoxP3^−^T cells in these three groups. (**F**) Relationship of the percentage of CXCR5^+^CD4^+^CD25^−^FoxP3^−^ T_FH_ cells and the fluorescent units of IgG-specific autoantibodies in the responder group. This experiment was repeated three times with 6-7 mice for each group and each plot represents one mouse in each group. Data shown were the mean ± SEM. *p < 0.05; ***p < 0.001; ns, no significance.

In order to investigate the potential T_FH_-associated differences in AIHA mouse model, transfused recipients were grouped as either non-responder (no autoantibodies) or responder (more than 75% erythrocyte with red cell-specific autoantibodies).The percentage of CD4^+^CXCR5^+^CD25^−^Foxp3^−^ T_FH_ cells was analyzed by flow cytometry in these groups. As shown in Fig. 2A–C, the percentage and number of CD4^+^CXCR5^+^CD25^−^Foxp3^−^ T_FH_ cells in the responder group were significantly increased compared with the non-responder and control groups. The proportion of ICOS^hi^PD-1^hi^ cells was also higher in the responder group than that of the non-responder and control groups (Fig. 2D,E). Further analysis found that there was a moderate and positive correlation between the percentage of CD4^+^CXCR5^+^CD25^−^Foxp3^−^ T_FH_ cell and autoantibody fluorescence intensity in the responder group (Fig. 2F).

### Expression of CD4+CXCR5+FoxP3+ cells in AIHA mouse model

**Figure 3:**
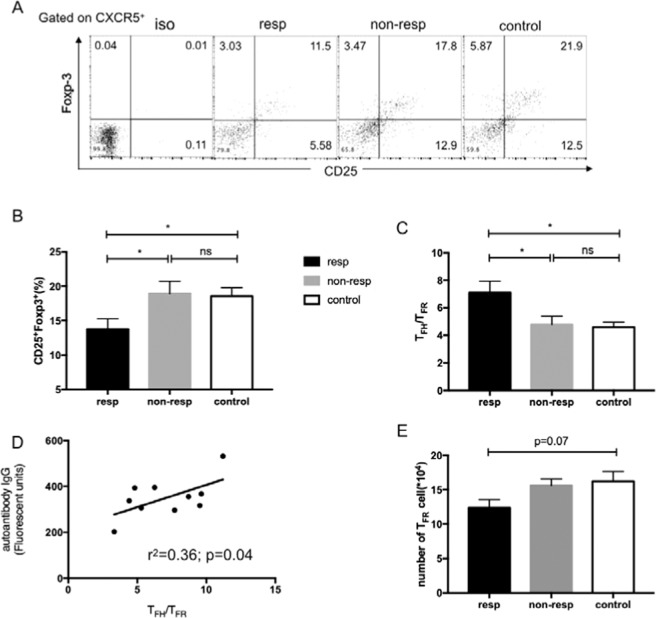
Decreased T_FR_ cells in autoantibody-positive AIHA mice. (**A**) The expression of CD25 and Foxp3 in CXCR5^+^CD4^+^T cells from mice in the responder, non-responder, and control groups. (**B**) The percentages of CD25^+^Foxp3^+^ cells in CXCR5^+^CD4^+^T cells in these three groups. (**C**) The ratio of T_FH_:T_FR_ in these three groups. (**D**) Relationship of T_FH_:T_FR_ and the fluorescent units of IgG-specific autoantibodies in responder the group. (**E**) The number of CD4^+^CXCR5^+^CD25^+^FoxP3^+^T_FR_ cells in spleen in these three groups. Experiments were repeated three times with 3–4 mice for each group. Data shown were the mean ± SEM. *p < 0.05; ns, no significance.

T_FR_ cells share features of both T_FH_ and Treg cells, localize to B-cell follicle, and regulate the size of the T_FH_ cell population and antibody response *in vivo*. So we tested whether increased T_FH_ population and autoimmune response in the AIHA mouse model were because of the shrunken T_FR_ subset. As shown in Fig. 3A and B, the percentage of CD25^+^ and Foxp3^+^ cells within the CD4^+^CXCR5^+^ T subset was lower in the responder group than that of the non-responder and control groups.The ratio of T_FH:_T_FR_ was higher in the responder group, and also, this ratio had a moderate and positive correlation to autoantibody fluorescence intensity in the responder’s group (Fig. 3C,D). Further analysis pointed out that the number of T_FR_ cells slightly decreased in the responder group (Fig. 3E), suggesting the decreased proportion of T_FR_ cell was because of expanded T_FH_ cells.

### Serum IL-4, IL-6 and IL-21 levels in the AIHA mouse model

**Figure 4:**
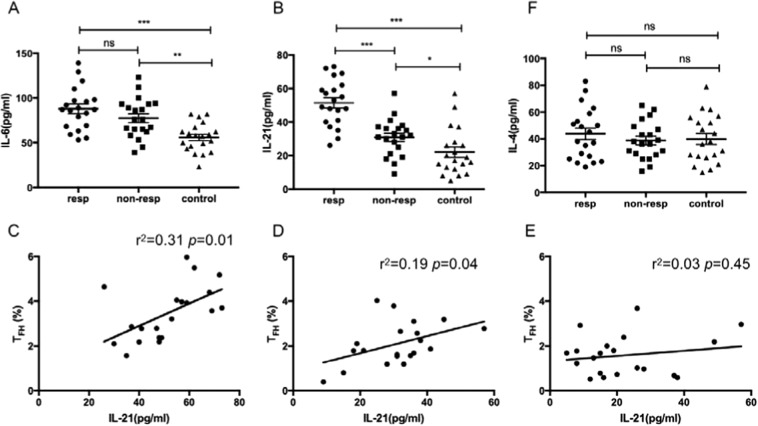
Relative cytokine levels in the AIHA mouse model. (**A**) Levels of serum IL-6 in the responder, non-responder, and control groups in the 12 weeks after the first immunization. (**B**) Levels of serum IL-21 in these three groups in the 12 weeks after the first immunization. (**C**–**E**) Relationship of serum IL-21 levels and the percentage of CXCR5^+^CD4^+^CD25^−^ T_FH_ cells in these three groups, respectively. (**F**) Levels of serum IL-4 in these threegroups in the 12 weeks after the first immunization. Each plot represents one mouse in each group. This experiment was repeated three times with 6–7 mice in each group. Data shown were the mean ± SEM. *p < 0.05; **p < 0.01; ***p < 0.001; ns, no significance.

Recent studies^16,28^ have indicated that the cytokines IL-6 and IL-21 play important roles in the differentiation and function of T_FH_ cells and in response to antibodies production. The serum IL-6 level was higher in both responder and non-responder groups compared to the control group, regardless of the presence of RBC autoantibodies (Fig. 4A). For serum IL-21 level, it was 2-fold higher in the responder group than the control group (Fig. 4B). The previous studies^29,30^ have demonstrated that the levels of both IL-21 and IL-6 are significantly associated with the frequency of T_FH_ cells in the autoimmune diseases. In the present study, serum IL-21 level was strongly and positively correlated with the percentages of CXCR5^+^CD4^+^CD25^−^Foxp3^−^T_FH_ cells in the responder group (Fig. 4C). Besides, there was a moderate and positive correlation between IL-21 level and CXCR5^+^CD4^+^CD25^−^Foxp3^−^ cells in the non-responder group and no significant correlation was found in the control group (Fig. 4D,E). No predictive relationship between serum IL-6 level and this parameter in all the three groups was found (data not shown). Besides IL-21, IL-4 is another cytokine secreted by T_FH_ cell and no difference was found among three groups (Fig. 4F).

### Bcl-6, c-Maf, and IL-21 mRNA expression in the AHIA mouse model

**Figure 5:**
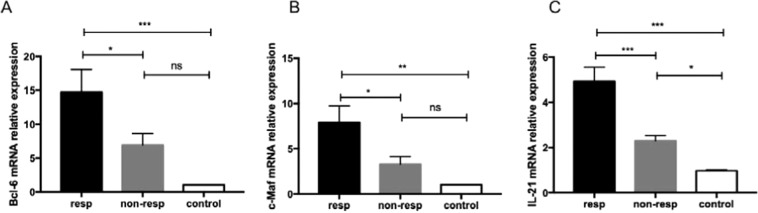
Expression of Bcl-6, c-Maf, and IL-21 mRNA in Tcells of the AIHA mouse model. (**A**) Levels of the relative expression of Bcl-6 mRNAof T cells from mice in the responder, non-responder, and control groups. (**B**) Levels of the relative expression of c-Maf mRNA of CD4^+^ T cells from these three groups. (**C**) Levels of the relative expression of IL-21 mRNA of CD4^+^T cells from these three groups. Experiments were repeated three times with 3–4 mice in each group. Data shown were the mean ± SEM. *p < 0.05; **p < 0.01; ***p < 0.001.

The transcriptional factors of Bcl-6 and c-Maf as well as cytokines IL-21 play crucial roles in the generation, differentiation, and function of T_FH_ cells^11^. The mRNA expression of Bcl-6, c-Maf, and IL-21 was assessed in these three groups, respectively, which were notably higher in the responder group than the control group (Fig. 5).

### CXCR5^+^CD4^+^CD25^−^T_FH_ cells play a positive role in the process of AIHA

**Figure 6:**
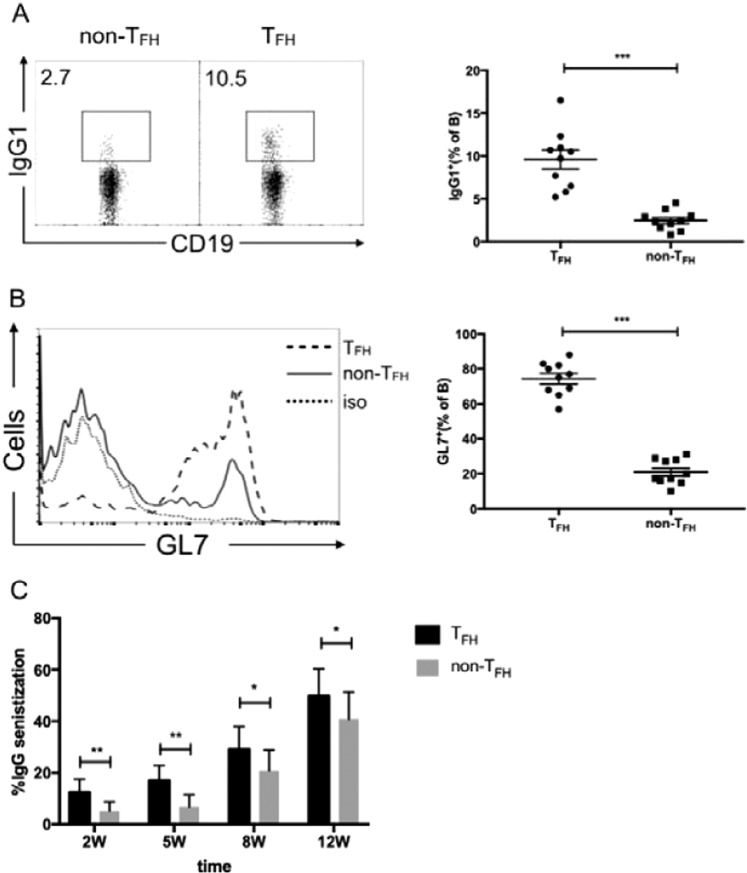
The role of T_FH_ cells in antibody productionand pathogenesis of AIHA. (**A**,**B**) CXCR5^+^CD4^+^CD25^−^T_FH_ cells and CXCR5^−^CD4^+^CD25^−^Tcells (non-T_FH_) sorted from the responder group in week12 were cultured with CD19^+^ B cells from similarly immunized mice along with anti-CD3 and anti-IgM for six days. B cells were intracellularly stained for IgG1 (**A**) and surface stained for GL7 (**B**). Plots were pre-gated on CD19^+^ B cells. Each plot represented a single well. Data were from four independent experiments and the mean ± SEM were shown. ***p < 0.001. (**C**) The sorted CXCR5^+^CD4^+^CD25^−^T_FH_ cells and CXCR5^−^CD4^−^CD25^−^T cells from the responder group were adoptively transferred into C57BL/6J mice one day before rat RBCs injection on a weekly basis. Level of IgG-specific autoantibodies on mouse RBCs was measured by flow cytometry and expressed as a percentage of background unstained cells on week 2, 5, 8, and 12. Data shown are of three independent experiments with five mice in each group.

To study whether an increased proportion of CXCR5^+^CD4^+^CD25^−^T_FH_ cells plays a role in the AIHA mouse model promotion activity, the *in vitro* B cell class switch recombination assays were investigated as described earlier^31^. CD4^+^CXCR5^+^CD25^−^T_FH_ and CD4^+^CXCR5^-^CD25^−^ T cells were sorted from the responder mice. These cells were cultured with CD19^+^ B cells (also isolated from the responder group) separately along with anti-IgM and anti-CD3. As expected, a significant increase in the promotion of class-switched IgG B cell by CD4^+^CXCR5^+^CD25^−^ T_FH_ cells compared to CD4^+^CXCR5^−^CD25^−^T cells was found (Fig. 6A). Next, expression of GL7 was examined as it is a sensitive marker for B cell activation in GC in these assays^32^. GL7 expression was increased 3- to 4-fold on B cells cultured with T_FH_ cells than that cultured with CD4^+^CXCR5^−^CD25^−^ T cells (Fig. 6B).

Further, the T_FH_ cell function was investigated *in vivo*. CD4^+^CXCR5^+^CD25^−^T_FH_ or CD4^+^CXCR5^−^CD25^−^T cells from the responder group were adoptively transferred into naive C57BL/6J mice. The day after transfer, the mice were immunized with rat RBCs weekly for consecutive 12 weeks. As demonstrated in Fig. 6C,autoantibodies can be detected as early as the second week in the mice adoptively transferred T_FH_ cells, whereas almost no autoantibody-positive red blood cells were detected in the mice transferred CD4^+^CXCR5^−^CD25^−^ T cells. This elevated autoantibody level continued until 12 weeks.

### The promotion capacity of T_FH_ cells between responder and non-responder groups

**Figure 7:**
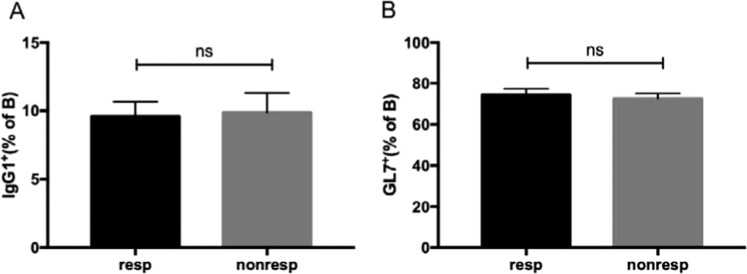
The equal functionality of T_FH_ cells between the responder and non-responder. CXCR5^+^CD4^+^CD25^−^T_FH_ cells sorted from the responder group and non-responder group in week 12 was cultured with CD19^+^ B cells from the responder group along with anti-CD3 and anti-IgM for six days. B cells were intracellularly stained for IgG1 and surface stained for GL7. The percentage of IgG1^+^(**A**) andGL7^+^ (**B**) cells was calculated in CD19^+^ B cells. Data were from three independent experiments and the mean ± SEM were shown. ns, no significance

Furthermore, we compared the ability of T_FH_ cells in the responder and non-responder groups by co-culturing the CD4^+^CXCR5^+^CD25^−^ T_FH_ cells from these two groups with CD19^+^ B cells along with anti-IgM and anti-CD3. As shown in Fig. 7, the expression of IgG1 and GL7 was similar in these two groups. Therefore, the promotion capacity of T_FH_ cells was not significantly altered in the responder and non-responder groups (Fig. 7).

## Discussion

Here, the number of CD4^+^CXCR5^+^CD25^−^ T_FH_ cells were increased in autoantibody-positive AIHA mouse, resulting in a high ratio of T_FH:_T_FR_. Besides, the transcription level of Bcl-6 and c-Maf and serum IL-21 was elevated concomitantly. Furthermore, increased T_FH_ cell activity was associated with the response against successive immunization with RBCs. Taken together, the results have significant implications on the role of T_FH_ cells in the pathogenesis of AIHA.

AIHA is a severe and sometimes fatal disease. Although lots of knowledge is known about the generation of the destructive effects of pathogenic autoantibody in AIHA, there is still much to learn about the influencing factors of antibody generation. Several mechanisms have been studied to contribute to AIHA, including dysregulation of central and peripheral tolerance mechanisms, disruption of cytokine axes, and molecular mimicry between autoantigens and pathogens^33–37^.

Most studies of AIHA focused on the erythrocyte-specific autoreactive B cells, while T cell tolerance was considered to be a stopgap to autoimmunity^38^. In 2005, with Playfair and Marshall–Clarke model, Amina^39^ found the importance of CD25^+^regulatory T subsets in controlling AIHA in C57BL/6J mice. Treatment with anti-CD25 antibody prior to immunization increased the incidence of AIHA from 30% to 90%. Intriguingly, Richards AL^40^ demonstrated that Tregs are non-essential components of tolerance to the HOD RBC autoantigen. Different results were probably attributed to different mouse model and gene background. Besides, it is reported that T helper 17 (Th17) cells could affect the development of AIHA by enhancing the adaptive humoral responses in AIHA patients and mouse models^41^. But until now, limited reports have been found about the T_FH_ and T_FR_ cells in the pathogenesis of AIHA.

By weekly intraperitoneal injection of rat RBCs into mice, erythrocyte autoantibodies were detectable within 5–6 weeks after immunization. The number of erythrocyte autoantibodies peaked in 10–12 weeks and correlated with a significant increased level of reticulocytes and a shortened RBC lifespan. These index parameters demonstrated that our AIHA mouse model was well-constructed. According to our results, the number of CD4^+^CXCR5^+^CD25^−^ T_FH_ cells was significantly increased in the responder group than that in non-responder and control groups, with the incremental expression of ICOS and PD-1 correspondingly. Consistently, the transcription factors Bcl-6 and c-Maf were highly expressed in the responder group. Furthermore, the increasing T_FH_ cell was moderately positively correlative associated with the anti-RBC IgG fluorescent units. It has been well known that interaction of T_FH_ cells with B cells in the GC plays a fundamental role in the differentiation of plasma cells and production of high-affinity antibodies^11^. From the co-culture experiment, the CD4^+^CXCR5^+^CD25^−^ T_FH_ cells, rather than CD4^+^CXCR5^−^CD25^−^ T cells, could promote B cell activation and antibody secretion. The adoptive transfer assay also confirmed the promotion function of T_FH_ cells because of the earlier onset and increased level of erythrocyte autoantibody in the AIHA mice with adoptive transfer CD4^+^CXCR5^+^CD25^−^ T_FH_ cells. The function of T_FH_ cells in the responder group was not altered compared to the non-responder group, despite the increased number of T_FH_ cells.

Interactions of autocrine or paracrine cytokines with the receptors provide essential signals for the differentiation and function of T_FH_ cells. Among them, IL-6 and IL-21 are most famous and well researched. IL-6, which is mainly secreted by the macrophage, can transiently induce the expression of the transcription factor Bcl-6 and IL-21, creating a positive feedback loop for enforcing the T_FH_ cell fate^16^. Hence, the early programming of T_FH_ cells is abated in the absence of IL-6. Although lack of IL-21 or IL-21 receptor did not affect the initial differentiation and expansion of T_FH_ cells, those T_FH_ cells failed to support GC reaction, leading to diminished levels of plasma cells and serum IgG. So, IL-21 is required for the T_FH_ cell persistence and function^17,28,42^. Note that IL-21, the main and vital cytokine secreted by T_FH_ cell, also influences B cell proliferation, survival and isotype switch, providing the bidirectional promotion role for both B cell and T_FH_ cell^15,43^. According to our research, serum IL-6 and IL-21 levels were significantly higher in the responder group than the control, and, more remarkable, serum IL-21 level was strongly and positively correlated with the percentages of CXCR5^+^CD4^+^CD25^−^T_FH_ cells in the responder group. Hence, the elevated IL-21 level was in favor of T_FH_ cell proliferation and function, leading to the excessive GC response and antibody secretion. Apart from IL-21, IL-4 is another major help molecule produced by T_FH_ cells to keep GC B cells alive and class switch recombination. However, no difference in serum IL-4 level was found between the responder and the control groups. Taken together, serum IL-21 level plays an important role in the T_FH_ function of AIHA.

Newly reported Treg subset T_FR_ cells could suppress T_FH_ cells and GC B cells function by inhibiting cytokine IL-4/IL-21 production, preventing GL7 and B7-1 expression on B cells and limited class switch recombination^22^. Hence, we suspected that the enlarged T_FH_ cell proportion and autoantibody secretion were caused by the T_FR_ cells. In our research, the shrunken proportion of T_FR_ cells in the responder group was discovered, as evidenced by decreased CD25^+^FoxP3^+^ subset among CD4^+^CXCR5^+^ cells. Therefore, the ratio of T_FH_:T_FR_ was enlarged, leading to an imbalance of T_FR_ and T_FH_ cells. Moreover, the cell count was almost unchanged among three groups, indicating that T_FR_ may not be the reason for the increased level of autoantibody in AIHA (Fig. 3E). Besides, no difference was found in the T_FR_ function between responder and non-responder group *in vitro* (data not shown).

Some reports about CD4^+^CXCR5^+^CD25^−^T_FR_ cells are considered as the terminally-differentiated T_FR_ cells and retain the expression of Foxp3^+^ and suppressive molecules CTLA-4^44^. Current studies suggest that down regulation of CD25 is a marker of T_FR_ development. CD25^+^ T_FR_ regulates the interactions at the T-B border and travels through the follicle, whereas CD25^−^ T_FR_ is responsible for direct suppression in the GC itself. Compared to CD25^+^ T_FR_, CD25^−^ T_FR_ cells shift its gene expression signature more similar to T_FH_ cell, displaying a high level of Bcl-6, CXCR5, and PD-1 and a low level of Foxp3, Blimp1, PSGL1^45^. In this study, the CD25^−^ T_FR_ was only 3-5% in the AIHA mouse (Fig. 3A). Similar to CD25^+^ T_FR_ cell, the proportion of CD25^−^ T_FR_ cell was lower in responder and non-responder groups than that of control group. However, no difference was found in the absolute number among the three groups (data not shown). So, CD25^−^ T_FR_ may-be not a key point for erythrocyte autoantibody production in AIHA.

Up to now, plentiful research has demonstrated the key role of T_FH_ and T_FR_ cells in autoimmune diseases, such as systemic lupus erythematosus (SLE), rheumatoid arthritis (RA), and idiopathic thrombocytopenic purpura (ITP)^46,47^. ITP, similar to AIHA, is characterized by the increased platelet destruction by autoantibodies directed against platelet glycoproteins. It is reported that there is an increase in the proportion of circulating T_FH_ cells and spleen T_FH_ cells in the ITP patients, particularly in the anti-platelet antibody-positive patients. Plasma IL-21 level is also significantly increased in active ITP patients^29,30,48^. The above clinical findings are in accordance with our results. It should be highlighted that the frequency of circulating T_FH_ cells returns to normal after therapy in the newly diagnosed ITP patients, whereas children who fall in chronic ITP have a persistent increase in both circulating T_FH_ cells and serum IL-21 level^48^.

Limitations to this research are present. The role of T_FH_ and T_FR_ cells in differentiating anti-rat antibody vs. anti-mouse autoantibody responses need to be further studied. The situation of T_FH_ and T_FR_ cells in AIHA patients should also be studied thoroughly in the future. Overall, the studies for the first time have shed light on the important role of T_FH_cells in regulating anti-RBC autoantibody production during the pathogenesis process of AIHA. Although the role of the inflammatory environment in the increase in T_FH_ frequency could not be completely excluded, our data strongly suggest that T_FH_ cells participate in B cells differentiation and anti-RBC-antibody production. It is hoped that a greater understanding of T_FH_ and T_FR_ cells can result in promising therapeutic approaches against AIHA.

## Materials and Methods

These studies were carried out in accordance with the approved guidelines of Peking University Second Hospital. All study methods and experimental protocols were approved by Peking University Second Hospital.

### Animals

C57BL/6J (B6) mice were purchased from the Beijing Vital River Laboratory Animal Technology Co. Ltd and were housed in a specific pathogen-free barrier facility with restricted access. All care and handling of animals were performed according to the standard guidelines for the care and use of experimental animals in Peking University Second Hospital.

### Immunization regimen for induction of AIHA

Rat RBCs were purchased from Zhengzhou Bestgene biotech company (Henan, China) and adjusted to 10^9^ cell/mL. Female C57BL/6J mice between 8 and 10 weeks old were immunized weekly for 12 weeks through intraperitoneal injections with 2 × 10^8^ rat RBCs in 200 μL RPMI.

### Detection and measurement of auto- and alloantibodies

Blood samples (25 μL) by retro-orbital sinus bleeding were obtained on a weekly basis, five days after each immunization. IgG sensitization autoantibodies levels on the RBCs were determined by flow cytometry using FITC–conjugated anti–mouse IgG (Invitrogen, Life Technologies, Grand Island, NY). For analysis of rat RBC-specific xenoantibodies, rat RBCs were incubated with diluted mouse plasma for one hour at 37 °C and after several washes, were stained with FITC-conjugated anti–mouse IgG as previously described^39^.

### Mouse RBCs survival studies and reticulocyte counts

Mouse RBCs(1 × 10^9^) were obtained from naive female C57BL/6J mice, labeled with PKH-26 (Sigma-Aldrich, St. Louis, MO) and injected by the tail-vein into control mice and those that had developed AIHA. Blood samples were obtained by retro-orbital sinus bleeding at the time points indicated after transfusion and the clearance of fluorescent RBCs was measured by flow cytometry as previously described^49^. Reticulocyte counts were performed using the Advia 120 Hematology System (Bayer, Tarrytown, NY).

### Flow cytometry and cell sorting

Single-cell suspensions were prepared from the spleen and the erythrocytes were depleted with the ACK lysis buffer. For surface staining, cells were incubated for 30 min at 4 °C with fluorescent-labeled monoclonal Ab specific for mouse CD4, CD8, CXCR5, CD25, GL7, B220 (BD Biosciences, San Jose, CA), PD-1 and ICOS (Invitrogen). For intracellular staining of Foxp3, cells stained with surface marker antibodies were fixed, permeabilized with Cytofix/Cytoperm (BD Biosciences, San Jose, CA) and incubated with APC conjugated anti-mouse Foxp3 (BD Biosciences) according to the manufacturer’s protocol. For intracellular staining of IgG1, cells were first fixed and permeabilized with Cytofix/Cytoperm (BD Biosciences) and then incubated with PE-conjugated anti-IgG1 (Biolegend, San Diego, CA). Corresponding isotype-matched control monoclonal antibodies were used in all flow cytometric staining procedures. Flow cytometric analysis was performed on FACSCalibur using CellQuest software (BD Biosciences). CD4^+^CXCR5^+^CD25^−^ T_FH_ or CD4^+^CXCR5^−^CD25^−^ T cells from mice in the responder and non-responder group were sorted using FACSAria II sorter cytometer (BD Biosciences).

### Enzyme-Linked Immunosorbent Assay (ELISA)

Serum from control mice and AIHA was used to test for the presence of cytokines IL-21, IL-6 and IL-4 with an enzyme-linked immunosorbent assay (ELISA; BioLegend, San Diego, CA). Each step was performed according to the manufacturer’s protocol.

### Quantitative mRNA Determinations

Total RNA was prepared from freshly isolated spleen CD4^+^T cells (5 × 10^6^) with TRIzol reagent (Invitrogen) and was used to make cDNA using random primers and the Reverse Transcription System (Promega, Madison, WI). For quantitative real-time PCR, iQ SYBR Green Supermix (Bio-Rad Laboratories, Hercules, CA) was used according to the manufacturer’s instructions. Quantitative PCR was performed on an iCycler (Bio-Rad Laboratories). The quantity of IL-21, Bcl-6 and c-Maf was normalized to the housekeeping gene Gapdh for each sample. The amplification conditions were as follows: 5 min at 95 °C for denaturation, and then 40 cycles at 95 °C for 10 s and 60 °C for 40 s. The fluorescence values were collected at 60 °C. The primer pairs used for PCR were as following: IL-21 Sense: 5-TCATCATTGACCTCGTGGCCC -3; Reverse: 5- ATCGTACTTCTCCACTTGCAATCCC -3; Bcl-6: Sense: 5-CACACCCGTCCATCATTGAA-3; Reverse: 5-TGTCCTCACGGTGCCTTTTT-3; c-Maf: Sense: 5-AGCAGTTGGTGACCATGTCG-3; Reverse: 5-TGGAGATCTCCTGCTTGAGG-3; Gapdh Sense: 5-CCTGGAGAAACCTGCCAAGTAT-3 Reverse: 5-AGAGTGGGAGTTGCTGTTGAAG-3.

### Cell culture

For T_FH_ stimulation assays, 2 × 10^4^ CD4^+^CXCR5^+^CD25^−^ T_FH_ cells and CD4^+^CXCR5^−^CD25^−^ T cells from mice of the responder group were plated with 5 × 10^4^ CD19^+^ B cells (all purified from spleen of responder group) and 2 μg/mL soluble anti-CD3 (BD Biosciences) plus 5 μg/mL anti-IgM (Jackson Immunoresearch, West Grove, PA). Cells were harvested and analyzed 6 days later.

### Adoptive transfer studies

CD4^+^CXCR5^+^CD25^−^ T_FH_ or CD4^+^CXCR5^−^CD25^−^ T cells from mice of the responder group were isolatedusing FACS Aria II sorter cytometer (Becton Dickinson). Approximately 2 × 10^4^ sorted CD4^+^CXCR5^+^CD25^−^ T_FH_ or CD4^+^CXCR5^−^CD25^−^ T cells in 0.1 mL of phosphate-buffered saline (PBS) was injected intravenously into 10-week-old female C57BL/6J recipient mice followed by weekly injections of rat RBCs one day later.

### Statistical analysis

Data are expressed as means ± SEM. One-way ANOVA analysis of variance was applied to determine whether an overall variation existed with statistical significance among the groups. Unpaired and paired Student’s t test was appropriately chosen to compare differences between two groups. The correlation between the two groups was analyzed by linear regression. Two-way repeated measures ANOVA was used for repeated measurement variables. P values of less than 0.05 were considered statistically significant.

## Data Availability

Any data of this study were available to the public if necessary.
